# Catechol inhibits epidermal growth factor-induced epithelial-to-mesenchymal transition and stem cell-like properties in hepatocellular carcinoma cells

**DOI:** 10.1038/s41598-020-64603-2

**Published:** 2020-05-06

**Authors:** Won-Chul Lim, Hyunhee Kim, Young-Joo Kim, Bu-Nam Jeon, Hee-Bum Kang, Hyeonseok Ko

**Affiliations:** 1 Korea Food Research Institute, Wanju-gun, Jeollabuk-do Republic of Korea; 2 Department of Biomedical Sciences, Asan Medical Center, AMIST, University of Ulsan College of Medicine, Seoul, Republic of Korea; 3 Natural Products Research Center, Korea Institute of Science and Technology, Gangneung, Gangwon-do Republic of Korea; 4 Genome and Company, Pangyo-ro 253, Bundang-gu, Seongnam-si, Gyeonggi-do Republic of Korea; 5 Voronoi Research Institute, S 12th F, Songdogwahak-ro 32 (IT center), Yeonsu-gu Incheon, Republic of Korea; 6 HLB Life Science, Teheran-ro 420, Gangnam-gu, Seoul, Republic of Korea

**Keywords:** Biochemistry, Cancer, Cell biology

## Abstract

Epithelial-mesenchymal transition (EMT) is a major cellular process in which epithelial cells lose cell polarity and cell-cell adhesion and become motility and invasiveness by transforming into mesenchymal cells. Catechol is one of the natural compounds present in fruits and vegetables and has various pharmacological and physiological activities including anti-carcinogenic effects. However, the effects of catechol on EMT has not been reported. Epidermal growth factor (EGF) is one of the growth factors and is known to play a role in inducing EMT. The present study showed that catechol suppressed not only the morphological changes to the mesenchymal phenotype of epithelial HCC cells, but also the reduction of E-cadherin and the increment of Vimentin, which are typical hallmark of EMT. In addition, catechol suppressed EMT-related steps such as migration, invasion, anoikis resistance acquisition, and stem cell-like characterization through the EGFR-AKT-ERK signaling pathway during liver cancer metastasis. Therefore, these results suggest that catechol may be able to regulate the early metastasis of liver cancer *in vitro*.

## Introduction

Hepatocellular carcinoma (HCC) is commonly prevalent cancer and their incidence and mortality rates are steadily rising in recent years^1^. It is most closely linked to chronic viral hepatitis infection exposure to alcohol or aflatoxin, type 2 diabetes, and obesity^2^. Currently, advanced therapeutic methods including surgical intervention for HCC were developed, however the lowest survival rate remains due to characteristics of late diagnoses, a lack of effective treatment methods, and easy metastasis in most HCC^3^. Therefore, exploring effective anti-HCC drugs and therapeutic strategy are necessary for to improve the survival rate of the HCC patients.

Epithelial-mesenchymal transition (EMT) is a cellular process that involves growth and differentiation required for normal embryonic tissue development. Recently, several studies have been reported that EMT in tumor cells is an important step in cancer progression, including tumor growth, invasion, and metastasis, and contributes to a high malignancy stage^4^. The key features of EMT is the change in epithelial cells to phenotypes, which is due to decreased adhesion between cells or cells and matrix, and cellular polarity, which leads to increased proliferation, and become migration and invasion of mesenchymal cells. When EMT occurs in cancer cells of primary tumors, cells undergo morphological and molecular changes, especially the expression of E-cadherin, ZO-1, occluding, and α-catenin, which are intercellular adhesion proteins, is decreased and N-cadherin, Vimentin, α-SMA, and fibronectin^5^. In addition, the molecular changes during EMT are caused by the regulation of transcription factors such as Snail, Twist, and ZEB families. These transcription factors are well known as E-cadherin repressors, which act by binding to E-box DNA sequence in E-cadherin promoter, result in activating the expression of Vimentin that promote cell motility and invasiveness^4^. In addition, previous studies have shown that EMT-mediated cell motility and invasive capacity are enhanced by several secretory factors from cancer cells, including MMPs, which are involved in the tumor invasion, metastasis, and angiogenesis in various cancer. MMP-2, gelatinase A/type IV collagenase, is which degrades cell surface proteins, including E-cadherin and extracellular matrix components, including gelatin, to invade into adjacent tissue through the basement membrane^6,7^. On the other hand, epithelial cells have the characteristic of anoikis and are designed to die when they come off the extracellular matrix, preventing cancer cells from growing on anchorage-independent cells or adhering to an inappropriate matrix and inhibiting colonization on other organs. Thus, an anoikis resistance, one of the EMT features, gives the ability of a migrating or invasive cancer cell to metastasize successfully to distant organs along the blood or lymph vessels. These overall EMTs and their associated early metastasis-related processes are activated by several growth factors such as transforming growth factor β (TGF-β), epidermal growth factor (EGF), platelet-derived growth factor (PDGF), and hepatocyte growth factor (HGF)^8^.

EGF is a ligand capable of binding to EGF receptor (EGFR) and regulates cell growth, differentiation, migration, invasion, and tumorigenesis in various cancer types, including liver cancer. EGFR, a member of the receptor tyrosine kinase (RTK) family, is frequently presented in HCC patients, and the activation of the EGF/EGFR signaling pathway is closely related to aggressive, intrahepatic metastasis, and poor clinical outcome^9^. Activation of EGFR by EGF leads to activation of several intracellular signaling pathways including Ras-Raf/mitogen-activated protein kinase pathway (MAPK)/extracellular signal-regulated kinase (ERK) pathway and phosphatidylinositol 3-kinase (PI3K)/AKT pathway^10^. According to some clinical studies, hepatocellular carcinoma patient infected with hepatitis C virus showed higher activation of AKT and ERK and lower survival rates than those without hepatitis C virus, thus these results revealed that not only expression of EGFR but also activation of AKT and ERK were could be used as a predict marker for HCC^11^. EGFR mediated AKT and ERK activation are known to be key pathways that regulate cell migratory ability and invasiveness through increased expression or activity of MMPs^10,12^. In addition, activation of AKT and ERK signaling pathways is a common mechanism by which cancer cells acquire anoikis resistance, which is associated with the suppression of proapoptotic protein expression and the promotion of anti-apoptotic protein expression^13^.

Cancer stem cells (CSCs), also known as tumor initiating cells, differ from normal cancer cells in that they represent tumorigenesis through self-renewal and differentiation. Recently, the characteristics and metastasis of CSC are known to be controlled by EMT as well as micro- and hypoxic environments^14,15^. In some studies, the EGF/EGFR signaling pathway has been known to contribute significantly to the formation and maintenance of CSCs in various cancer. CD44 and Nanog belong to several regulators of CSCs and are abnormally high in CSC and can be used as markers for CSC and it is known that their expression is increased by EGF signaling pathway in several cancers including HCC^16,17^. The purpose of present research is to understand the suppressive effect of catechol and its molecular mechanism on EMT induced by EGF in human HCC *in vitro*. We found that catechol suppresses Vimentin expression, the mesenchymal marker, while at the same time increases E-cadherin expression, the epithelial cell marker, in the EGF-treated HCC cells. In addition, catechol has been shown to reduce the levels of EMT-mediated migration, invasion, anoikis resistance, and stem cell-like properties through the inhibition of EGFR/AKT/ERK signal cascade activation during metastasis. These results revealed that catechol *in vitro* inhibits EMT and stem cell-like properties in human hepatocellular carcinoma cells, indicating its potential to be used as anticancer drugs.

## Results

### Catechol inhibits cell proliferation of Huh7 and PLC/PRF/5 cells

**Figure 1:**
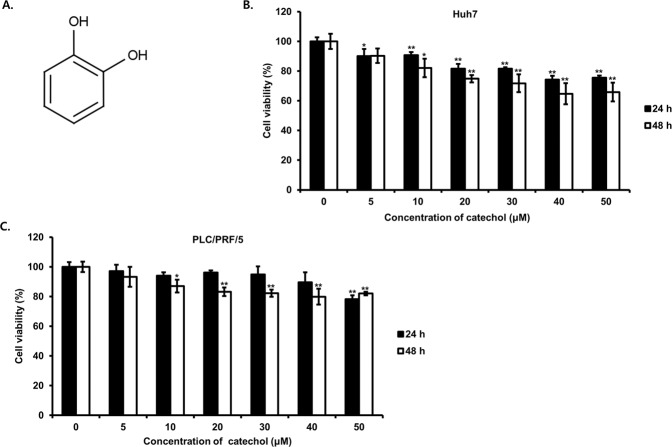
Inhibitory effect of catechol on the proliferation in Huh7 and PLC/PRF/5 hepatocellular carcinoma cells. (**A**) The chemical structure of catechol is presented. (**B,C**) The changes of cell proliferation treated with catechol at concentrations of 0, 5, 10, 20, 30, 40, and 50 μM for 24 or 48 h were measured by CCK-8 assay. **p < 0.01, *p < 0.05 *versus* EGF-untreated cells. Values are represented as means ± SD for independent experiments performed in triplicate.

To investigate whether catechol (Fig. 1A) inhibits proliferation of HCC cells, we measured changes of cell proliferation in HCC cells by treatment of catechol at a variety concentration (0, 5, 10, 20, 30, 40, and 50 μM) during 24 or 48 h and cell viability was examined by WST-8 assay. WST-8 reacts with mitochondrial dehydrogenase of viable cells to produce water soluble formazan product. Also, WST-8 assay is higher detectable than the other tetrazolium salts-based assays. As results, viability of HCC cells was decreased dose-dependently by treatment of catechol for 24 or 48 h, however 5 and 10 μM concentrations of catechol were appeared above 80% cell proliferation than that of DMSO treated control cells (Fig. 1B,C). Therefore, 5 and 10 μM concentrations of catechol were selected as non-influence to anti-proliferation of HCC cells for further experiments.

### Catechol inhibits EGF-induced EMT of Huh7 and PLC/PRF/5 cells

**Figure 2:**
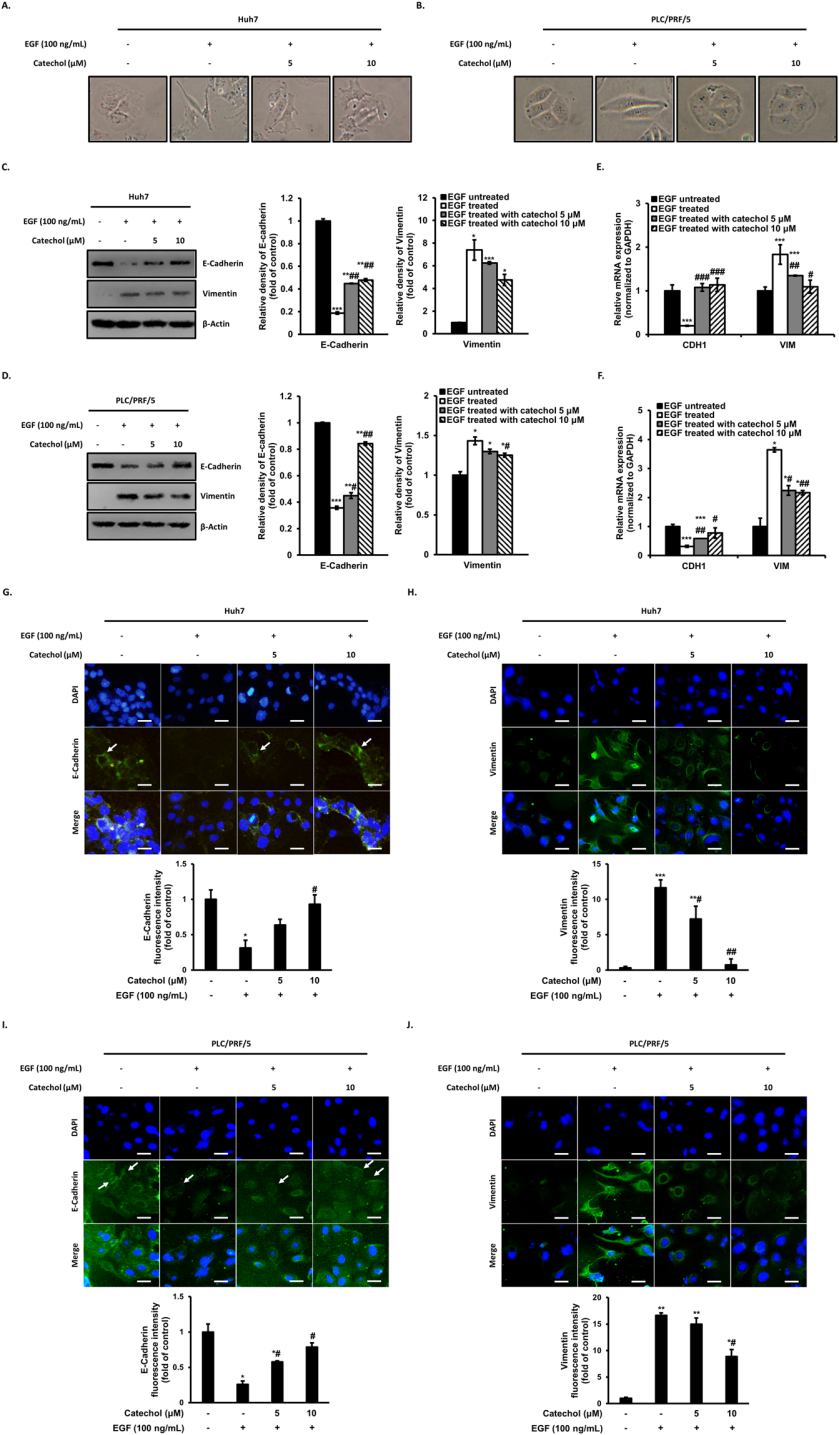
Catechol inhibits EMT by EGF of Huh7 and PLC/PRF/5 cells. These cells were treated with indicated concentration of catechol and stimulated with EGF for 48 h. The epithelial cell phenotypes of EGF-untreated (**A**) Huh7 and (**B**) PLC/PRF/5 cells (tight and round shape) were changed to elongated and mesenchymal morphology by EGF treatment. However, catechol prevented EGF-induced morphological changes from epithelial to mesenchymal and maintained a near-epithelial shape even though EGF was treated. (**C,D**) Expression and (**E,F**) transcription levels for epithelial marker E-cadherin and mesenchymal marker Vimentin were measured by Western blot, densitometric analysis, and quantitative real-time PCR, respectively. β-Actin was used as a loading control. (**G–J**) Immunolocalization of E-cadherin and Vimentin were observed by Immunofluorescence staining (scale bar: 20 μm). ***p < 0.0005, **p < 0.005, *p < 0.05 *versus* EGF-untreated cells and ###*p* < 0.0005, ##*p* < 0.005, #*p* < 0.05 *versus* EGF-treated control. The data are expressed as mean ± SD for triplicates. Original blotting images used in Figs. 2C and 2D are presented in Fig. S3.

EMT process is characterized molecular alteration of EMT markers including E-cadherin and Vimentin, followed by occurring morphological changes enable to cell migration. In prior to measuring the EMT inhibitory activity of catechol in hepatocellular carcinoma cells, the expression changes of EMT biomarkers through various growth factor treatments were determined. As a result, it was confirmed that EGF changed the expression of EMT biomarkers including E-cadherin and vimentin most remarkably (Fig. S1), and further the suppressive effect of catechol against EMT by EGF was conducted. To investigate whether catechol inhibits EMT by EGF, morphology of HCC cells was observed using inverted light microscopy. Huh7 and PLC/PRF/5 cells were treated with EGF (100 ng/mL) with or without catechol at the indicated concentrations for 48 h, it was observed that HCC cells progressed from epithelial morphology to mesenchymal phenotype containing elongated and spindle-like shapes via EGF treatment. However, treatment of catechol inhibited morphological changes by EGF, suggesting catechol prevents morphological changes to mesenchymal phenotype as an evidence of underwent EMT in HCC cells (Fig. 2A,B). EGF also has been shown to reduce E-cadherin expression and increase Vimentin expression in a variety types of tumor cells^18^. As results of Western blotting analysis, EGF stimulation notably decreased the protein level of E-cadherin, whereas it notably increased that of Vimentin compared with control cells, and these alterations were dose-dependently inhibited through catechol treatment (Fig. 2C,D). Moreover, similar with the protein levels, the mRNA level of E-cadherin was reduced and that of Vimentin was increased by EGF treatment, however these EGF-induced transcription levels of E-cadherin and Vimentin were attenuated by catechol treatment (Fig. 2E,F). Furthermore, the expression of E-cadherin in cell membrane and cytoplasm was decreased by EGF treatment whereas catechol suppressed the decrease of E-cadherin expression (Fig. 2G,I). However, Vimentin, founded in the cytoplasm of mesenchymal, was increased by EGF treatment compared with EGF-untreated cells whereas catechol decreased the increase of Vimentin expression (Fig. 2H,J). Therefore, these data revealed that catechol could suppresses the EMT induction by EGF in HCC cells.

### Catechol suppresses Snail expression by EGF

**Figure 3:**
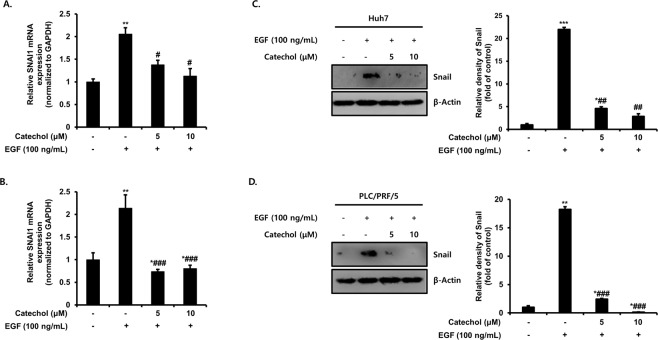
Catechol suppresses Snail, an EMT transcription factor by EGF. Huh7 and PLC/PRF/5 cells were treated with only EGF or EGF plus catechol for 48 h. (**A,B**) The transcriptional level of Snail was analyzed by quantitative real-time PCR. Fold change was calculated by 2^−∆∆Ct^ relative quantitative analysis. (**C,D**) Expression of Snail protein was measured by Western blot and densitometric analysis. β-Actin was used as a loading control. **p < 0.005, *p < 0.05 *versus* EGF-untreated cells and ###*p* < 0.0005, ##*p* < 0.005, #*p* < 0.05 *versus* EGF-treated cells. These data are expressed as mean ± SD for triplicates. Original blotting images used in Figs. 3C and 3D are presented in Fig. S4.

Snail (encoded by *SNAI1*) is a transcription factor containing zinc finger ion that has been established as an E-cadherin repressor (encoded by *CDH1*) and an EMT stimulator. It certainly represses E-cadherin expression, which is the hallmarks of EMT and is considered to suppress tumor progression^19^. To investigate the effects of catechol on Snail expression by EGF, we measured the levels of mRNA and protein expressions by qRT-PCR, Western blotting, and densitometric analysis, respectively. As results, EGF notably increased the mRNA level of Snail by about two-folds whereas catechol significantly decreased that of Snail in HCC cells (Fig. 3A,B). Similarly, increase protein level of Snail by EGF was also down-regulated by catechol treatment (Fig. 3C,D). Therefore, these data showed that catechol suppresses E-cadherin expression through inhibiting Snail by EGF in HCC cells.

### Catechol prevents migration and invasion through inhibition of EGF-induced MMP-2 expression and activity

**Figure 4:**
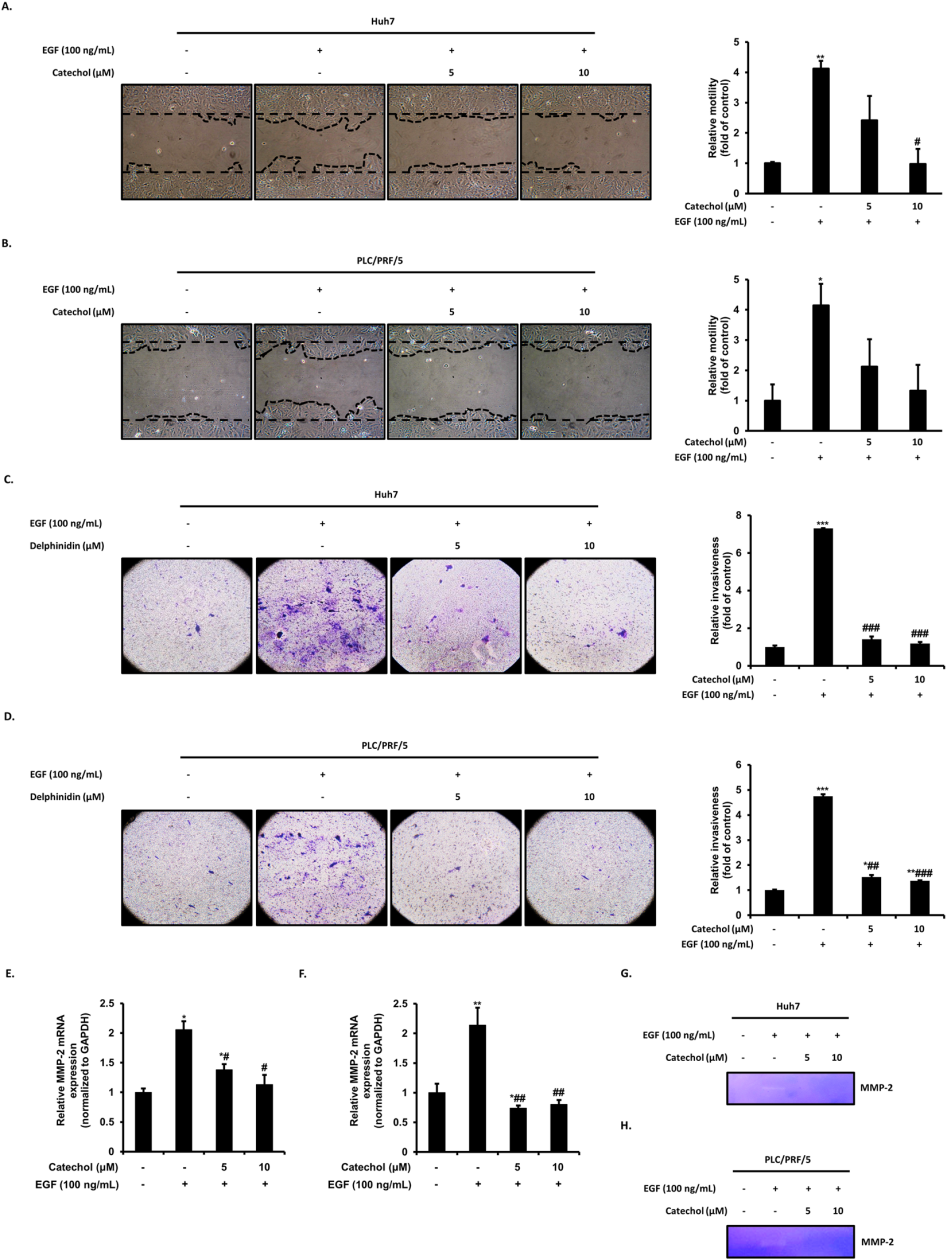
Catechol inhibits migration and invasion by EGF of Huh7 and PLC/PRF/5 cells. (**A,B**) Catechol suppressed the wound healing by EGF during 48 h. The wound healing area of the cells were photographed and quantified by Image J program. (**C,D**) Catechol suppressed the invasive ability by EGF during 48 h. *In vitro* invasiveness of Huh7 and PLC/PRF/5 cells were measured by staining invaded cells that infiltrated through the membrane containing extracellular matrix layer. (E-H) Effect of catechol on mRNA expression and activity of MMP-2 activated by EGF. (**E,F**) The transcriptional level of MMP-2 was measured by quantitative real-time PCR. Fold change was calculated by 2^−∆∆Ct^ relative quantitative analysis. (**G,H**) Huh7 and PLC/PRF/5 cells were treated with only EGF or EGF plus catechol in serum-free medium for 48 h. The media supernatant were harvested and used for analyze MMP-2 activity by gelatin zymography. The MMP-2 activity of the EGF-untreated cells was set to 1 and based on this, the relative MMP-2 activity was expressed as a fold changes. The data are expressed as mean ± SD for triplicates. ***p < 0.0005, **p < 0.005, *p < 0.05 *versus* EGF-untreated cells and ###*p* < 0.0005, ##*p* < 0.005, #*p* < 0.05 *versus* EGF-treated cells. Original zymogram images used in Figs. 4G and 4H are presented in Fig. S5.

Numerous findings indicated that the EMT is required for metastatic cascade during malignant progression including cell motility and invasiveness^20,21^. First, to measure whether catechol inhibits cell migration of EGF-induced HCC cells, a Wound healing assay was employed by scratching cell monolayer prior to stimulation of EGF 100 ng/mL with or without catechol at concentrations of 5 and 10 μM. After treatment of the EGF with or without catechol for 48 h, representative migrated cell area was photographed and calculated by Image J program (NIH, Bethesda, MD). As results, EGF significantly stimulated motility by 4-folds compared with untreated cells in HCC cells, whereas catechol dose-dependently suppressed the increase cell migration ability by EGF (Fig. 4A,B).

Secondary, to evaluate suppressive effect of catechol against cell invasion by EGF in HCC cells, a Matrigel invasion assay was employed using Boyden chamber in which the top side of the 8 μm-pore membrane is coated with one of three gel layers: basement membrane, collagen I, and laminin. HCC cells were pretreated with EGF in presence or absence of catechol at concentrations of 5 and 10 μM for 48 h prior to incubate in an invasion inner chamber. The pretreated cells were transferred to inner chamber and incubated with EGF plus catechol or not for 48 h. The infiltrated cells, located on bottom surface of the membrane by infiltrating ECM-like membrane, were stained, photographed, and analyzed by measuring OD at 570 nm. As results, EGF-stimulated cells were appeared higher cell invasion ability by 7.3-folds in Huh7 or 4.8-folds in PLC/PRF/5 cells than that of untreated cells. However, catechol treatment exerts lower cell invasion ability than that of EGF-treated cells (Fig. 4C,D).

According to experimental evidence, EGF has been known to improve cell motility and invasiveness by MMP-2 activation, results in cancer cells enable cross several ECM barriers during tumor metastasis^22–24^. As results, MMP-2 mRNA expression of HCC cells was significantly up-regulated by EGF stimulation. In contrast, catechol treatment significantly decreased the EGF-induced mRNA level of MMP-2 (Fig. 4E,F). Subsequently, to verify the role of catechol on MMP-2 activity of HCC cells, Zymography assay was employed to measure whether catechol inhibits proteolytic activity against stimulation of EGF in HCC cells. As data shown in Fig. 4G,H, MMP-2 gelatinolytic activities of HCC cells were increased by EGF stimulation whereas, these activities were diminished by catechol treatment. These results were consistent with the data about inhibitory effects of catechol against motility and invasiveness of HCC cells. Taken together, catechol could suppress MMP-2 activity and its transcription level by EGF, results in decreases EGF-induced cell migration and invasion in HCC cells.

### Catechol prevents anoikis resistance and reattachment through inhibition of EGF-stimulated EMT

**Figure 5:**
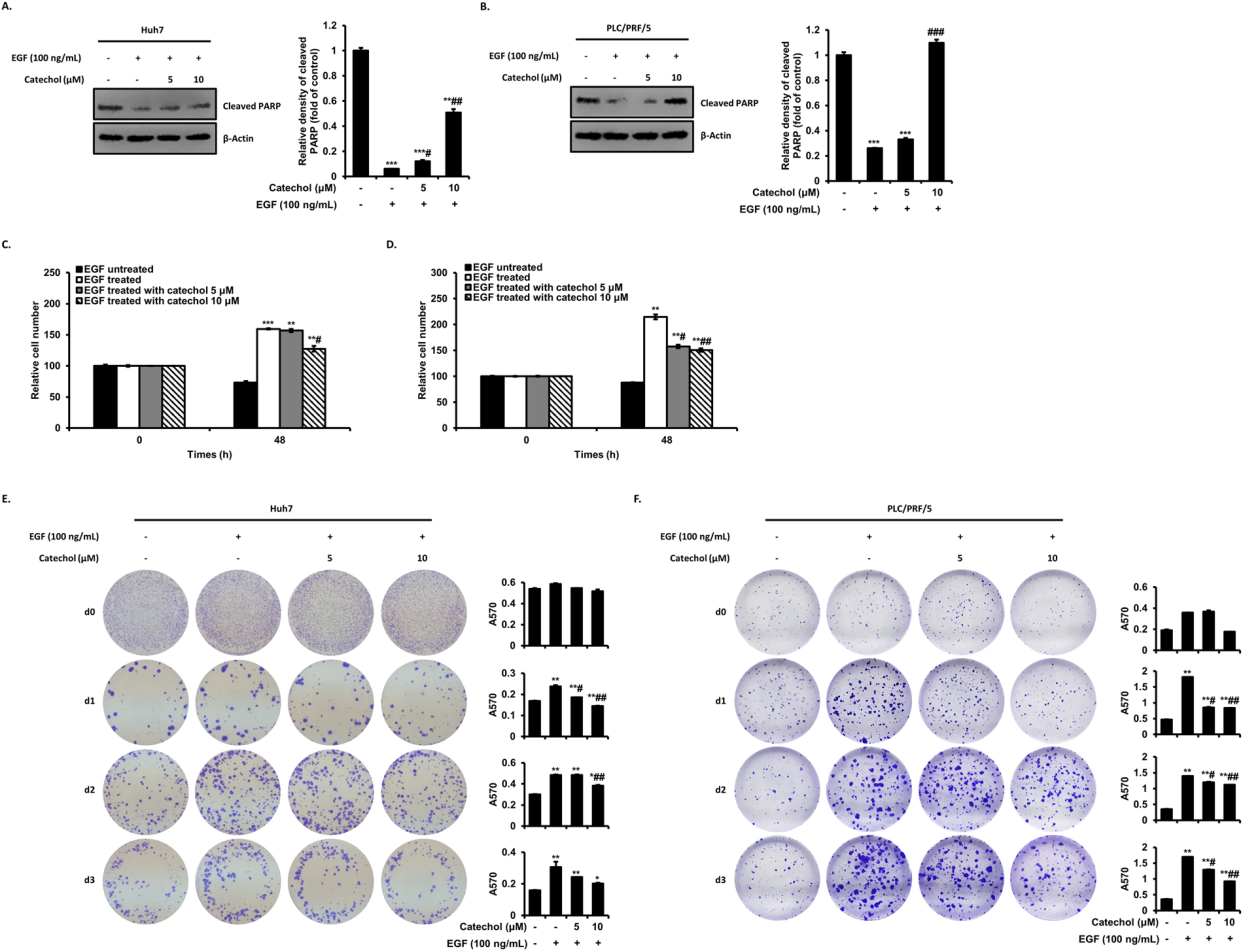
Catechol inhibits EGF-induced anoikis resistance and reattachment. Huh7 and PLC/PRF/5 cells were pretreated with only EGF or EGF plus catechol for 48 h, and then these cells were cultured with only EGF or EGF plus catechol in anchorage-independent condition using poly-HEMA-coated tissue culture plates for 48 h. (**A,B**) The cleavage of PARP was measured by Western blot and densitometric analysis. (**C,D**) The amounts of the Huh7 and PLC/PRF/5 cell suspensions were investigated by SRB assay. The cell number of the EGF-untreated cells was set to 100 and based on this, the relative cell number was expressed as a percentage. (**E,F**) The HCC cells were grown in suspension for 0 to 3 d and subsequently transferred into adhesive plates followed by Crystal violet staining were performed. Reattachment of cells resistant to anoikis was analyzed by absorbance measurements of stained cells. These data were shown as mean ± SD of three independent experiments. ***p < 0.0005, **p < 0.005, *p < 0.05 *versus* EGF-untreated cells and ###*p* < 0.0005, ##*p* < 0.005, #*p* < 0.05 *versus* EGF-treated cells. Original blotting images used in Figs. 5A and 5B are presented in Fig. S6.

Anoikis is a cell death that is occurred upon anchorage-independent condition. Anoikis resistance of tumor cells is representative property of the EMT and is required for tumor cells successfully metastasize through blood circulation. Also, EGFR activation by its ligands suppresses anoikis in various cancer^13^. Hence, we examined the apoptotic property of HCC cells in the suspended condition using poly-HEMA plates. HCC cells began to anoikis after 48 hours, whereas EGF stimulation prevents the anoikis of HCC cells. In contrast, catechol increased anoikis of HCC cells even though treatment of EGF. As results, EGF treatment reduced the cleavage of PARP, which is occur by anoikis, whereas the decrease expression was significantly inhibited by catechol treatment (Fig. 5A,B). We also measured inhibitory effects of catechol against EGF-induced anoikis resistance in HCC cells by SRB assay. This method relies on the stoichiometric binding of SRB dye to proteins under mild acidic conditions and its subsequent extraction under basic conditions. The amount of dye extracted is a proxy for cell mass and thus the number of cells in a sample. As results, HCC cells appeared cell death in anchorage-independent condition for 48 h, whereas EGF treatment exerted anoikis resistance, which is significantly prevented by catechol treatment (Fig. 5C,D). Subsequently, crystal violet staining was performed to measure whether catechol suppresses reattachment by anoikis resistance of the cells that grown in suspended conditions for the indicated time points. Several studies showed that improved resistance to apoptosis in anchorage-independence could contribute to the increased reattachment observed in modified anoikis assay^25^. As shown in Fig. 5E,F, EGF treatment increased reattach ability compared with untreated, whereas catechol treatment significantly inhibited increase the ability by EGF during 3 d. These results suggest that catechol suppresses the enhance anoikis resistance and reattachment of suspended HCC cells by EGF through preventing cleavage of PARP. Taken together, catechol could have potential roles in preventing EGF-induced anoikis resistance and reattachment of HCC cells through inhibiting of EGF-induced EMT.

### Catechol suppresses stem cell-like properties by EGF

**Figure 6:**
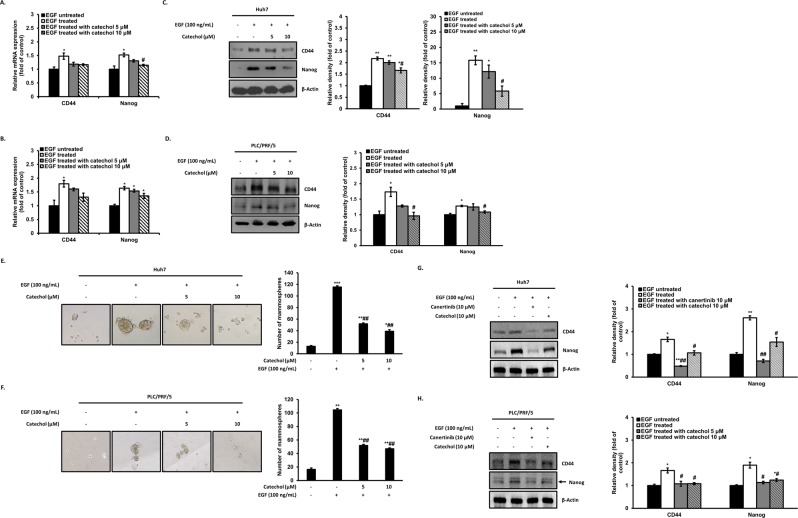
Catechol inhibits EGF-induced stem cell-like properties. Huh7 and PLC/PRF/5 cells were treated with only EGF or EGF plus catechol or canertinib for 48 h. (**A,B**) The mRNA levels of stem cell markers, including CD44 and Nanog, were analyzed by quantitative real-time PCR (n = 3). Fold change was calculated by 2^−∆∆Ct^ relative quantitative analysis. (**C,D**, **G,H**) Protein expressions of CD44 and Nanog were measured by Western blotting and densitometric analysis and the relative expressions were normalized to β-Actin. (**E,F**) The number of mammosphere formed by stem cell-like properties in suspended condition for 7 d was counted through a phase contrast microscope. ***p < 0.0005, **p < 0.005, *p < 0.05 *versus* EGF-untreated cells and ##*p* < 0.005, #*p* < 0.05 *versus* EGF-treated cells. The data are expressed as mean ± SD for triplicates. Original blotting images used in Figs. 6C, 6D, 6G, and 6H are presented in Fig. S7.

Recently, investigation of EMT in hepatocellular carcinoma has been newly approach by analyzing of circulating tumor cells (CTC) which is more likely to metastasis because it causes more aggressive and malignant phenotypes, this evidence be able to provide a more varied problem of HCC. Also, several studies have shown that EGF-EGFR signaling pathway plays important roles in maintenance, proliferation, and self-renewal of cancer stem cells^16^. Numerous attempts have been made to identify HCC cells with stem cell properties enriched some cell surface phenotype, specially CD44 and Nanog as cancer stem cell markers. Therefore, we employed quantitative real-time analysis to measuring transcription levels of stem cell markers. As results, expressions of CD44 and Nanog mRNA were significantly increased by EGF treatment compared with those of untreated cells. Interestingly, the stem cell marker levels were dose-dependently reduced by treatment of catechol (Fig. 6A,B). Also, CD44 and Nanog protein levels were down-regulated by catechol (Fig. 6C,D). Subsequently, we performed a Mammosphere assay to determine the role of catechol on stem cell-like properties by EGF. We founded that EGF-stimulated HCC cells acquired higher number of mammosphere than untreated cells, whereas the increase number of mammosphere by EGF were decreased by catechol treatment (Fig. 6E,F). These results were consistent with suppressive effects of catechol against EGF-induced EMT. Canertinib is an irreversible tyrosine-kinase inhibitor with activity against EGFR enable to inhibit wound healing by AKT and ERK signaling pathways, which are downstream pathway of EGFR signaling pathway^26^. Therefore, canertinib, an EGFR antagonist, was used as a positive control in experiments. As shown in Fig. 6G,H, not only catechol but also canertinib at concentration of 10 μM decreased CD44 and Nanog by EGF. Therefore, catechol could inhibit stem cell-like properties by acting as an antagonist against EGF, an inducer of EMT.

### Catechol inhibits activation of EGFR, AKT, and ERK by EGF

**Figure 7:**
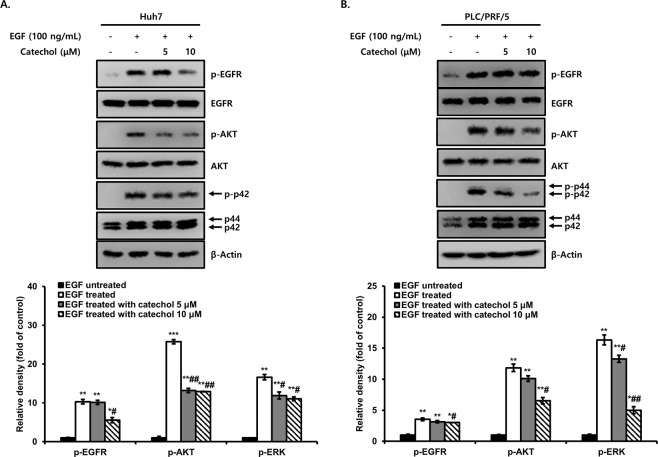
Effect of catechol on p-EGFR, p-AKT, and p-ERK expression activated by EGF. (**A**) Huh7 and (**B**) PLC/PRF/5 cells were treated with only EGF or EGF plus catechol for 1 h. The phosphorylation of EGFR, AKT, and ERK was analyzed by Western blotting and densitometric quantification and the relative phosphorylation was normalized to each total form. ***p < 0.0005, **p < 0.005, *p < 0.05 *versus* EGF-untreated cells and ##*p* < 0.005, #*p* < 0.05 *versus* EGF-treated cells. The data are expressed as mean ± SD for triplicates. Original blotting images used in Figs. 7A and 7B are presented in Fig. S8.

EGFR is a transmembrane protein that is activated by EGF ligands, which are leading to the stimulation of AKT and ERK signaling pathways controlling mainly migration, invasion, anoikis resistance, and stem cell-like properties during cancer progression^13^. To investigate whether catechol antagonizes against activations of EGFR, AKT, and ERK by EGF, HCC cells were pretreated with catechol 5 and 10 μM for 2 h in prior to stimulated with 100 ng/mL of EGF for 1 h, and p-EGFR, p-AKT, and p-ERK were detected by Western blotting and densitometric analysis. As results, p-EGFR, p-AKT, and p-ERK were significantly increased by treatment of EGF compared with those of untreated cells. However, when EGF treated with catechol at indicated concentrations in HCC cells, EGF-induced phosphorylation of EGFR, AKT, and ERK were dose-dependently decreased by catechol treatment (Fig. 7A,B).

## Discussion

Despite the evolution of HCC treatment techniques, surgical resection is still used to treat many patients with of HCC. This resection is not only associate with 5-year survival rate of 50%, but also with a recurrence rate of 70%. In addition, about 80% of HCC patients are diagnosed at a late stage because of the absence of specific symptoms and rapid progression^27^. During the metastasis, malignant tumor cells from solid tumors are separated by cell de-differentiation of the epithelial cells that gain migratory and invasive ability as well as decrease cell-cell junctions. The phenotypic transition of epithelial tumor cells was termed EMT and was confirmed in various cancer^28,29^. The early metastasis process can be described as follows: when EMT occurs in tumor cells, detachment from primary tumor takes place, followed by migration, invasion, anoikis resistance, and colonization in distant organs.

EMT is a transient and reversible process in which epithelial cells lose their cell polarity, cell-cell junction, and migration and invasion abilities are increased to become mesenchymal phenotype^30^. When epithelial cells enter the EMT process, morphological and molecular changes occur due to the down-regulate expression of the E-cadherin, ZO-1, and occludin and the up-regulate expression of N-cadherin, Vimentin, α-SMA, and fibronectin. Representative property of EMT is the functional diminution of E-cadherin, because several transcription factors such as Snail, Slug, ZEB1, and TWIST are associated with such EMT regulation during malignant tumor progression. In particular, the Snail protein belongs to the zinc finger containing Snail superfamily, which is known as a transcription repressor by linking strongly to the transcription start site of the E-cadherin gene. E-cadherin-deficient malignant tumor cell lines exhibit significantly higher expression of Snail, whereas E-cadherin-positive cells transfected with Snail represents stimulation of EMT and up-regulation of mesenchymal markers^31^.

EGF is one of the growth factors capable of binding to EGFR, and plays a major role in cell growth, migration, invasion, and metastasis, and is highly presented in many human cancer types. EGFR is commonly expressed in HCC and the EGFR signaling is closely related to aggressive tumors, metastasis, and poor clinical diagnosis. Activation of EGFR by EGF leads to the receptor dimerization, followed by activation of intrinsic tyrosine-specific kinase, occurs autophosphorylation of EGFR tyrosine kinase residues^32^. Several researches have been reported that EGFR signal pathway exerts disruption of desmosomes and adherens junctions leading to dissociation of cancer cells and improved cell migration, invasion, anoikis resistance, and stem cell-like properties. The interaction of EGFR and its ligands increases the MMP-2 activity, thereby enhancing cell motility and invasiveness. Some studies have shown that targeting of EGFR-EGF interaction by EGFR antibody-treated cells suppresses the expression of MMP-2 and its activity^33^. MMP-2 regulates migration and invasion during tumor metastasis via extracellular matrix protein degradation. It is frequently expressed in many types of carcinoma, including HCC, and its inhibition has been shown to suppress HCC intravasation, extravasation, and metastasis^34,35^. Clinically, overexpression of MMP-2 are considered to be involved in improved metastasis and poor diagnosis in liver cancer patients^36,37^.

Anoikis is an established apoptosis occurred immediately after detachment from adjacent cells or ECM, and is a key mechanism to prevent cell adhesion to adherence-independent cell proliferation and improper matrix. However, there are several mechanisms by which cancer cells fail to endure anoikis though detachment from the ECM, which resists anoikis, progresses to malignant tumors, and spreads widely to secondary sites. Tumor cells acquire anoikis resistance, which is due to the adaptation of the metastasis site to a specific through switching of their integrins, undergoing EMT, and the stimulation of pro-survival signaling cascade. The ability of the cancer cells to overcome the anoikis is acquired by EMT-mediated mesenchymal cell phenotype, which increases level of the anti-apoptotic gene, Bcl-2 and activation of the pro-survival signaling including AKT and ERK signal pathways through EMT-related factors including Snail, ZEB1/2, Twist, NF-κB, and HIF1/2. One of the important roles of EMT in cancer is contribute to formation of circulating tumor cells (CTCs), results in increasing tumor cell invasion, enhancing cell intravasation, and promoting cancer cell survival in the peripheral system. CTCs release tumor cells into the venous and/or lymphatic vessels, allowing them to circulate in the human body as a fountainhead of metastasis. Thus, CTCs have been provided information on prognosis, metastasis, treatment efficacy, and chemotherapy resistance in some types of cancers^38^.

Several researches have been reported that EMT is involved in stem cell characterization in cancer cells. Cancer stem cells (CSCs) are tumor cells that display characteristics related to normal stem cells, and have the ability to differentiate into all types of cancer found in particular cancer specimens. Therefore, CSCs are distinguished from other tumor cells by their ability to regenerate through self-renewal and multi-cellular differentiation. Thus, the development of specific therapies targeting CSCs could be improve the survival rate and recurrence rate of patients with metastatic cancer^39^. Other studies have been shown that CTCs induce the expression of CSCs phenotype, suggesting that EMT is closely associated with CTCs and CSCs, increasing the survival rate in the peripheral circulation^40^. Recent studies in prostate cancer cells have been shown that CSCs with EMT are resistant to radiation therapy through the PI3K/AKT/mTOR pathway. Immunohistochemical tests of patients with liver cancer showed increased numbers of AKT- or ERK-positive cells infiltrating lymph or blood vessels^39^. Thus, through a number of evidences, cancer cells could be found to undergo cancer metastasis through several steps such as migration, invasion, anoikis resistance, and stem cell properties by EMT.

As understanding of cancer metastasis has accumulated, many cancer therapies have been used. Recently, researches on natural compounds have been actively carried out, and they are attracting attention as materials for development of anti-cancer therapeutics having safety, low cost, and possibility of concurrent treatment. Catechol is also called as pyrocatechol or 1,2-dihydrobenzene that is structurally contained in flavonoids, phenolic compounds^41^. It is present in fruits and vegetables such as onions, apples, olive oil, and aronia^42^. Also, several studies documented that catechol existed in numerous medicinal herb such as *Rhus tripartitum*, *Bidens pilosa*, and *Gynostemma pentaphyllum*. It has been found that catechol or its moiety is present as a major component of various phenolic compounds in a lot of medicinal herbs, and exhibits such effect as anticancer, anti-inflammation and antioxidant^43–45^.

In this study, viability of HCC cells was not affected by treatment of catechol under concentration of 10 μM. In the *in vitro* and *in vivo* experiments of the previous studies, it was used at concentration of 100 μM in breast cancer and 10–40 μM in lung cancer, without affecting cell proliferation^42,46^. Thus, the catechol concentrations of 5 and 10 μM that does not affect cell proliferation was used. Our results showed that catechol inhibited the level of epithelial marker and increased the level of mesenchymal marker, and maintained the epithelial phenotype against EGF treatment, suggesting catechol inhibits EGF-stimulated EMT in HCC cells. Snail is known to inhibit the expression of E-cadherin via linking to between the E-box sequence in the promotor site of the E-cadherin gene and the C-terminal region including the zinc-finger of the Snail as a typical EMT-related transcription factor^47^. Our study showed that catechol treatment significantly increased the transcription and expression levels of E-cadherin, while also inhibiting the transcription and expression levels of Snail. High levels of Snail are known to induce metastatic phenotypes of cancer as well as reduced levels of E-cadherin. Some studies have shown that Snail levels are significantly higher in patients with metastatic HCC than in patients with non-metastatic HCC^48^. In this study, the results of catechol effectively inhibiting migration, invasion, anoikis resistance, and stem cell-like properties during early metastasis of hepatocellular carcinoma are consistent with the both transcriptional inhibited Snail and increased E-cadherin by catechol. For this reason, numerous studies have suggested that the reduction of Snail and the increment of E-cadherin are important as evidence for the inhibitory effect of candidates against EMT. Therefore, catechol seems to up-regulate the expression of E-cadherin by inhibiting the level of Snail during EGF-induced EMT. Some studies have been shown that quercetin, including the structure of catechol, increases E-cadherin expression and reduces Vimentin expression by reducing EGF-induced expression of Snail in prostate cancer^49^.

Previous studies have been shown that many natural compounds such as quercetin, resveratrol, and delphinidin inhibit motility and invasiveness via EGF-stimulated EMT suppression in several types of cancer cells, including HCC^49–51^. Likewise, catechol treatment inhibited migration and invasion in a dose-dependent manner during EMT by EGF in HCC cells. In addition, catechol decreased the activation of MMP-2 and phosphorylation of EGFR, AKT, and ERK in EGF-treated HCC cells treated with EGF and these results indicate that catechol could migrate and invade by modulating the activity of MMP-2 through the EGFR signaling pathway. These results are similar to prior findings that both expression and activity of MMP-2 by EGF inhibit motility and invasiveness by inhibition of AKT or ERK in fibrosarcoma cancer^52^. In addition, activation and overexpression of AKT and ERK have been known to cause anoikis resistance, one of the EMT characteristics. The present results reported that EGF-activated cells acquire anoikis resistance compared to untreated cells under anchorage-independent conditions through Western blotting, densitometric analysis, SRB assay, and anoikis assay. In contrast, catechol notably suppressed EGF-stimulated cell growth in suspension. These results suggested that catechol induces anoikis through suppressing EGF-stimulated EMT and activation of EGFR/AKT/ERK signaling cascade.

Cancer cells that spread during tumor metastasis require self-renewal ability consistent with stem cells for macroscopic metastatic progression and this ability is increased by EMT^53^. Moreover, in some findings, level of E-cadherin was reduced and level of Vimentin was increased in CSCs, which highly expressed CD44 and Nanog as CSCs markers. In particular, CD44 is known to produce mammosphere and tumors through interaction with EGFR^54^. In this study, the EGF-EGFR signaling pathway modulates the level of CSCs markers such as CD44 and Nanog, resulting in an increase in sphere formation under anchorage-independent conditions consistent with the content of other literature mentioned above.

In summary, we demonstrated that catechol inhibits EMT, migration, invasion, anoikis resistance, and stem cell-like properties through EGFR-AKT-ERK signaling pathways in EGF-treated human HCC cells. These results suggested the possibility of catechol as a potential candidate for preventing or treating HCC metastasis. However, the suppressive effect of catechol against HCC metastasis was not evaluated *in vivo*. Therefore, further experimentation using animal models are needed to verify the inhibitory role of catechol against HCC metastasis.

## Materials and Methods

### Reagents and antibodies

Catechol (1,2-dihydroxybenzene) was obtained from Sigma Aldrich (St. Louis, MO), prepared 100 mM stock solution in DMSO, and placed in dark at −20°C. Recombinant human epidermal growth factor (EGF) was purchased from Prospec (East Brunswick, NJ), dissolved at a concentration of 100 μg/mL in sterile water containing 0.1% bovine serum albumin (BSA), and placed in deep freezer at −70°C. Polyclonal anti-E-cadherin antibody was purchased from BD Biosciences (San Jose, CA). Polyclonal anti-Vimentin, anti-phospho-EGFR, anti-EGFR, anti-β-actin, anti-CD44, and anti-Nanog antibodies were obtained from Santa Cruz Biotechnology (Dallas, TX). Monoclonal anti-Snail, anti-phospho-AKT, anti-AKT, anti-phospho-ERK, anti-ERK antibodies were obtained from Cell Signaling Technology (Beverly, MA). Horseradish peroxidase-conjugated anti-mouse or anti-rabbit antibodies were obtained from Santa Cruz Biotechnology (Dallas, TX).

### Cell culture and drug treatment

Human hepatocellular carcinoma Huh7 and PLC/PRF cells were purchased from American Type Culture Collection (Manassas, VA). These cells were grown in RPMI-1640 medium (Hyclone, Logan, UT) containing 10% fetal bovine serum (FBS) and 1% penicillin-streptomycin (Hyclone, Logan, UT). Cell cultures were maintained at 37°C and 5% carbon dioxide in air. To induce EMT, EGF (100 ng/mL) was treated with RPMI-1640 complete media when cell confluence reached 30–40% in HCC cells. Catechol at different concentrations was also treated with EGF for 48 h.

### Assessment of cell proliferation

To investigate the cell viability, WST based Cell Viability Assay (EZ-cytox, Dogen Bio, Seoul, Korea) was employed. The assay procedure described as follows: Hepatocellular carcinoma cell lines (5 × 10^3^ cells/well) were seeded into 96-well plate with triplicates for each group and incubated at 37°C for overnight. On next day, a variety concentration of catechol was added into each cell line and incubated for 24 or 48 h. Control group was treated with 0.01% (v/v) dimethyl sulfoxide (DMSO) instead of catechol for 24 or 48 h. After incubation, cells were reacted with WST-8 10 μL per each well for 2 h and then, amount of formazan was detected by measuring absorbance at 450 nm using microplate reader.

### Western blot analysis

EGF or catechol were added into HCC cells and incubated for 48 h. After incubation, the cells were lysed in a RIPA buffer containing protease inhibitor cocktail (Roche Diagnostics, Mannheim, Germany). The quantified proteins by Bradford assay were denatured with 2× sample buffer at 95°C for 5 min and used as a loading sample for SDS-PAGE. A constant amount of protein (10–30 μg) was separated, transferred, and reacted with the primary antibody at 4°C for overnight. The membrane was washed and reacted with the secondary antibody for 1 h at room temperature. Then, washing step was performed three times for 5 min with TBST buffer, exposed to ECL solution (GE Healthcare, Buckinghamshire, UK), and visualized with a chemiluminescence reader (Vilber Lourmat, Collégien, France).

### RNA isolation and quantitative real-time PCR

Total RNA isolation from the HCC cells were performed using RNA isoPlus reagent (Takara Bio, Shiga, Japan). The procedure was performed according to the protocol of the manufacturer. Subsequently, quantified RNA was used for cDNA synthesis using cDNA Reverse Transcription kit (Applied biosystem, Carlsbad, CA). Real-time PCR was performed using Real-Time PCR 2× Master Mix (ELPIS Biotech, Daejeon, Korea), forward and reverse primers of target genes, and cDNA. The information on the primer sequence of the target genes is given as follows. E-cadherin: 5′-AAAGGCCCATTTCCTAAAAACC-3′ (forward) and 5′- TGCGTTCTCTATCCAGAGGCT-3′ (reverse); Vimentin: 5′-TGTCCAAATCGATGTGGATGTTTC-3′ (forward) and 5′-TTGTACCATTCTTCTGCCTCCTG-3′ (reverse); Snail: 5′-CCCCAATCGGAAGCCTAACT-3′ (forward) and 5′-GCTGGAAGGTAAACTCTGGATTAG-3′ (reverse); MMP-2: 5′-ACATCAAGGGCATTCAGGAG-3′ (forward) and 5′-GCCTCGTATACCGCATCAAT-3′ (reverse); GAPDH: 5′-CGCGGGGCTCTCCAGAACATCATCC-3′ (forward) and 5′-CTCCGACGCCTGCTTCACCACCTTCTT-3′ (reverse). The polymerase chain reaction was performed by heat treatment at 95°C for 1 min, followed by 40 cycles at 95°C for 30 s and 60°C for 1 min. The mRNA expression was normalized by comparing with the expression of GAPDH.

### Confocal immunofluorescence staining

Huh7 and PLC/PRF/5 cells (3 × 10^4^ cells/well) were seeded on 15-mm coverslips pre-placed in 24-well plates. On next day, EGF in presence or absence of catechol at concentrations 5 and 10 μM were treated to HCC cells for 48 h. The coverslips were washed with PBS (pH 7.4), fixed with 4% paraformaldehyde, and permeabilized with 0.1% Triton X-100 solution. Then these cells were blocked for 30 min in 1% BSA solution. After removed the blocking solution, the coverslips were reacted with indicated primary antibody for overnight at 4°C, which was followed by washed 5 times with PBST and incubated with secondary goat anti-mouse fluorescein isothiocyanate (FITC) conjugated antibody (Santa Cruz) for 1 h at room temperature. The coverslips were washed with PBST, counterstained and mounted with DAPI and mounting solution for 5 min. The immunolocalization of E-cadherin and Vimentin were examined using fluorescence microscope.

### Wound healing assays

HCC cells were seeded at an appropriate concentration to reach 90% cell confluency until the next day, and then scratched regularly using 200 μL pipette tip and cultured for 48 h with EGF (100 ng/mL) plus catechol or not. The area of the migrated cells was observed through a microscope and the representative location was photographed. The degree of cell migration was examined by ImageJ program.

### Matrigel invasion assays

Cell invasion kit containing transwell (Cell Biolabs, San Diego, CA) was employed and the procedure was carried out according to the protocol of the manufacturer. Briefly, EGF with or without catechol were pretreated for 48 h, and cells were recovered using trypsin/EDTA. The equal number of cells were seeded in Matrigel-coated transwells and then incubated with EGF and catechol for 48 h. After incubation, the remaining cells that did not pass through the membranes contained in the transwell were removed mildly. Only the cells invaded on the lower surface of the membrane were left, and stained with a cell staining solution for 10 min. The membrane was washed with tap water, and specimens were observed under a microscope. The dyed membrane was treated with 10% acetic acid solution to dissolve the dye. Optical density of the dye was measured at 560 nm to analyze the cell invasive ability.

### MMP gelatin zymography

HCC cells were incubated with EGF in presence or absence of catechol in serum-free medium for 48 h. After incubation, the culture medium containing MMP-2 was obtained and concentrated through Centrifugal Filter (Millipore, Temecula, CA). The concentrated supernatant was incubated with 2× sample buffer not containing β-mercaptoethanol for 5 min at RT and then separated by molecular weight through electrophoresis using 10% SDS-PAGE gel including 0.1% gelatin. Then, the gel was washed with 0.1% Triton X-100 solution three times for 5 min, renatured for 30 min, and developed at 37°C for overnight. The Coomassie blue staining was performed to identify the degraded substrate in the gel.

### Anoikis assays

HCC cells were pretreated with EGF in presence or absence of catechol at concentrations of 5 and 10 μM for 48 h, then these cells were detached and resuspended as single cell suspension with complete medium. Cell suspensions were transferred into poly-HEMA coated plate and cultured for 48 h. The floated cells were collected and media supernatants were removed by centrifuge at 10,000 rpm for 1 min. Cells were resuspended with fresh media, transferred to 24-well adhesive plates, cultured for overnight, and followed by cell survival of suspended cells was determined by Crystal violet staining. Briefly, cells were washed twice with PBS, fixed with formalin, and stained with crystal violet solution. The stained cells were washed, air-dried, photographed, and dissolved with 10% acetic acid, and optical density was measured at 570 nm.

### SRB assays

Protein content of suspended cells was examined by sulforhodamine B colorimetric (SRB) assay to measure cell proliferation. Briefly, after collection of cell suspension, medium supernatant was removed by centrifuge at 10,000 rpm for 1 min. Then, cells were fixed with ice-cold 10% trichloroacetic acid at 4°C for 30 min, washed five times with distilled water, and air-dried for 5 min. The dried cells were stained with 50 μL of 0.4% SRB solution in 1% acetic acid for 30 min. The stained cells were washed four times with 1% acetic acid, air-dried, and dissolved with 10 mM Tris solution (pH 10.5). After centrifugation, supernatants were collected and optical density at 570 nm was read using microplate reader.

### Mammosphere assays

For mammosphere assays, HCC cells were pretreated with EGF (Prospec, East Brunswick, NJ) in presence or absence of catechol at concentrations of 5 and 10 μM for 48 h, then pretreated cells were enzymatically harvested using trypsin-EDTA, resuspended as single cell suspension by pipetting, and transferred into poly-HEMA-coated plates to culturing in anchorage-independent condition. After 7 d, the representative of mammosphere was photographed and the number of mammosphere was counted under an inverted light microscope.

### Statistical analysis

All experiments were repeated at least three times and the results were expressed as mean and standard deviation. Statistical significance was analyzed using t-test. P value was considered to be significant within a range of less than 0.05.

## Supplementary information

Supplementary information.
