# Supplementary material for: Catechol inhibits epidermal growth factor-induced epithelial-to-mesenchymal transition and stem cell-like properties in hepatocellular carcinoma cells
Source: Sci Rep. 2020 May 6;10:7620. doi: 10.1038/s41598-020-64603-2 (PMC7203133; doi:10.1038/s41598-020-64603-2)
Supplement: Supplementary file 1 — Supplementary information. [file 41598_2020_64603_MOESM1_ESM.pdf]

Catechol inhibits epidermal growth factor-induced epithelial-to-mesenchymal transition and stem cell-like properties in hepatocellular carcinoma cells

Won-Chul Lim[1], Hyunhee Kim[2], Young-Joo Kim[3], Bu-Nam Jeon[4], Hee-Bum Kang[5], Hyeonseok Ko[6]*

[1]Korea Food Research Institute, Wanju-gun, Jeollabuk-do, Republic of Korea

[2]Department of Biomedical Sciences, Asan Medical Center, AMIST, University of Ulsan College of Medicine, Seoul, Republic of Korea

[3]Natural Products Research Center, Korea Institute of Science and Technology, Gangneung, Gangwon-do, Republic of Korea

[4]Genome and Company, Pangyo-ro 253, Bundang-gu, Seongnam-si, Gyeonggi-do, Republic of Korea

[5]Voronoi Research Institute, S 12th F, Songdogwahak-ro 32 (IT center), Yeonsu-gu, Incheon, Republic of Korea

[6]Biomedical Research Center, Asan Institute for Life Sciences, Seoul, Republic of Korea

*Corresponding author: Hyeonseok Ko, drug9054@naver.com

Keywords
Catechol, Epidermal growth factor (EGF), Epithelial-mesenchymal transition (EMT), Cancer stem cell (CSC), Hepatocellular carcinoma (HCC)

Supplementary Figure legends

Supplementary Figure 1. Changes of E-cadherin, N-cadherin, and vimentin by TGF-β1, HGF, and EGF in human Huh7 and PLC/PRF/5 hepatocellular carcinoma cells. Original blotting images used in Supplementary Figure 1 is presented in Supplementary Figure 2.

Supplementary Figure 2. Original western blot images used in Supplementary Figure 1.

Supplementary Figure 3. Original western blot images used in Figure 2C and 2D.

Supplementary Figure 4. Original western blot images used in Figure 3C and 3D.

Supplementary Figure 5. Original zymogram images used in Figure 4G and 4H.

Supplementary Figure 6. Original western blot images used in Figure 5A and 5B.

Supplementary Figure 7. Original western blot images used in Figure 6C, 6D, 6G, and 6H.

Supplementary Figure 8. Original western blot images used in Figure 7A and 7B.

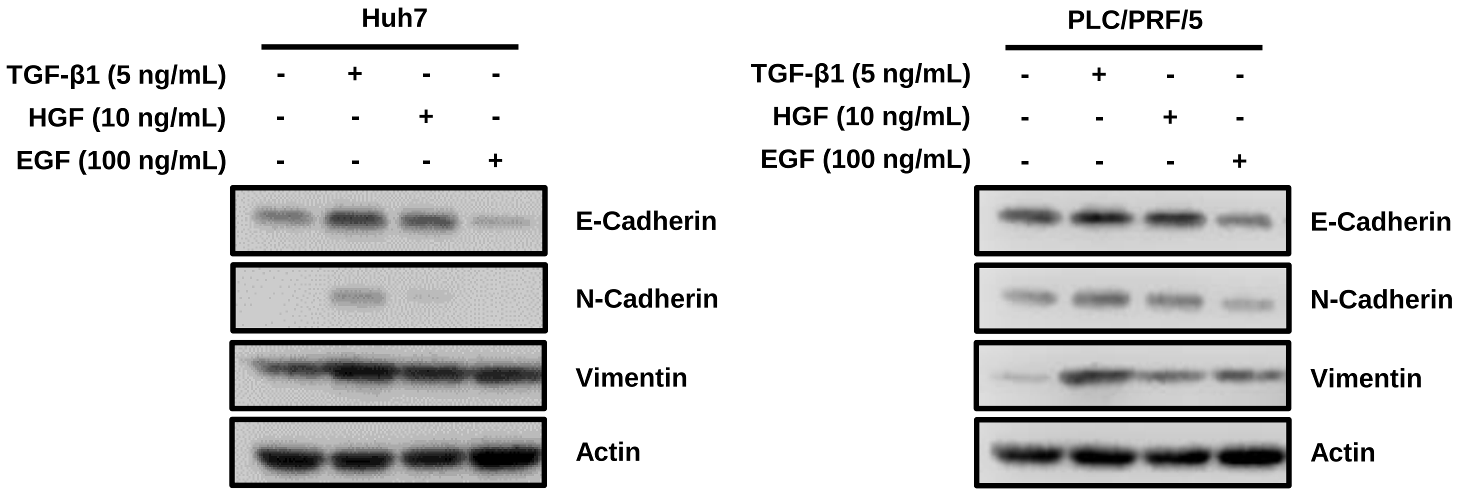
Huh7
TGF-β1 (5 ng/mL) - + - -
HGF (10 ng/mL) - - + -
EGF (100 ng/mL) - - - +
E-Cadherin
N-Cadherin
Vimentin
Actin
PLC/PRF/5
TGF-β1 (5 ng/mL) - + - -
HGF (10 ng/mL) - - + -
EGF (100 ng/mL) - - - +
E-Cadherin
N-Cadherin
Vimentin
Actin

Fig. S1

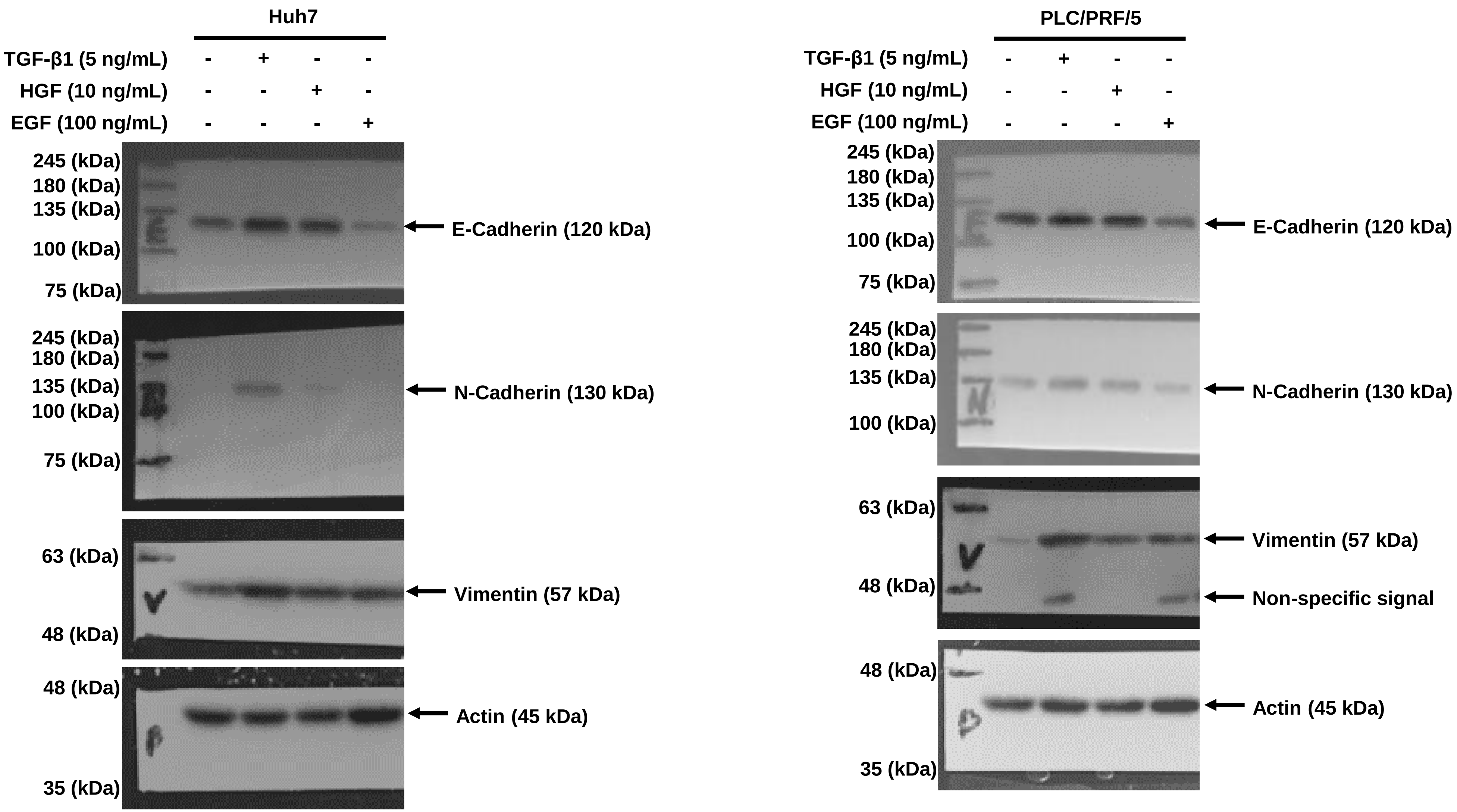
Huh7
TGF-β1 (5 ng/mL) - + - -
HGF (10 ng/mL) - - + -
EGF (100 ng/mL) - - - +
245 (kDa)
180 (kDa)
135 (kDa)
100 (kDa)
75 (kDa)
E-Cadherin (120 kDa)
245 (kDa)
180 (kDa)
135 (kDa)
100 (kDa)
75 (kDa)
N-Cadherin (130 kDa)
63 (kDa)
48 (kDa)
Vimentin (57 kDa)
48 (kDa)
35 (kDa)
Actin (45 kDa)
PLC/PRF/5
TGF-β1 (5 ng/mL) - + - -
HGF (10 ng/mL) - - + -
EGF (100 ng/mL) - - - +
245 (kDa)
180 (kDa)
135 (kDa)
100 (kDa)
75 (kDa)
E-Cadherin (120 kDa)
245 (kDa)
180 (kDa)
135 (kDa)
100 (kDa)
N-Cadherin (130 kDa)
63 (kDa)
48 (kDa)
Vimentin (57 kDa)
Non-specific signal
48 (kDa)
35 (kDa)
Actin (45 kDa)

Fig. S2

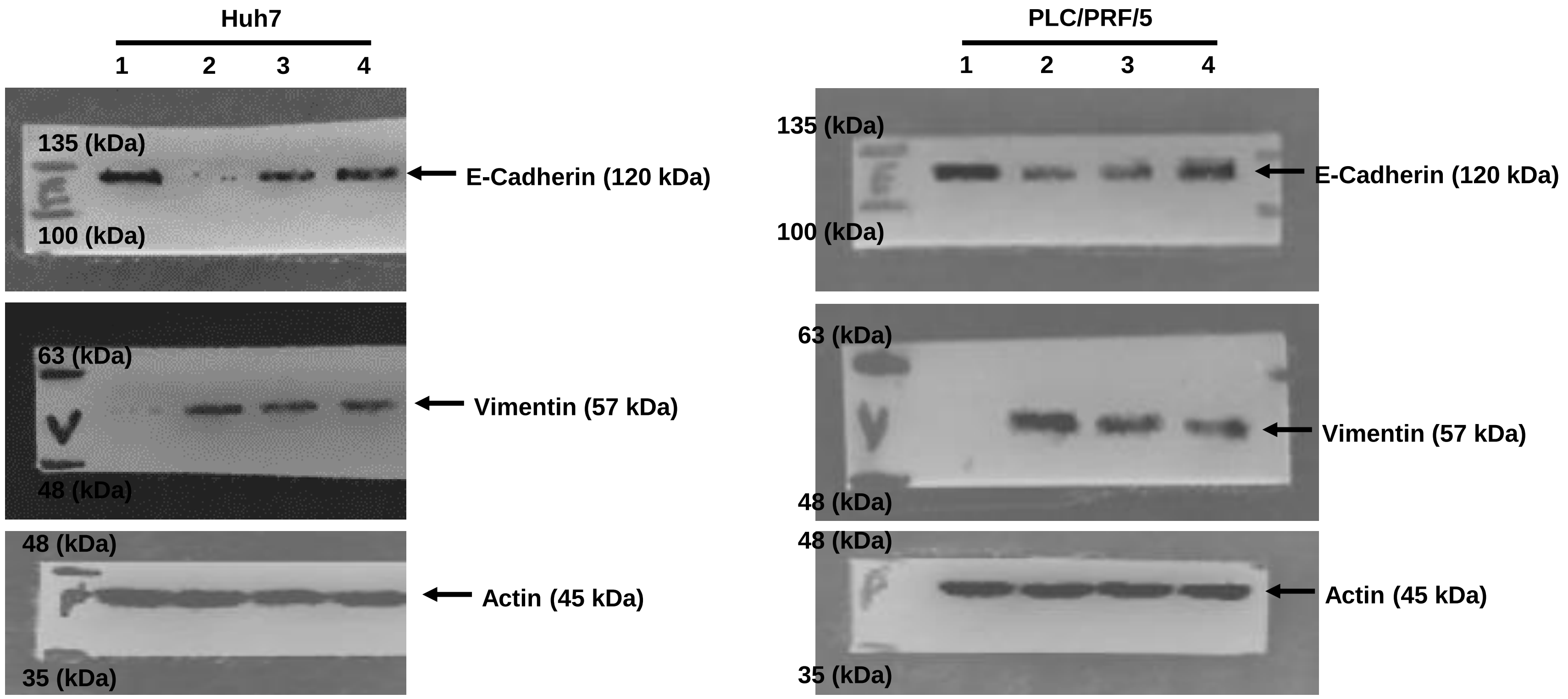

Legend
1: EGF untreated
2: EGF treated
3: EGF treated with catechol 5 μM
4: EGF treated with catechol 10 μM

Fig. S3

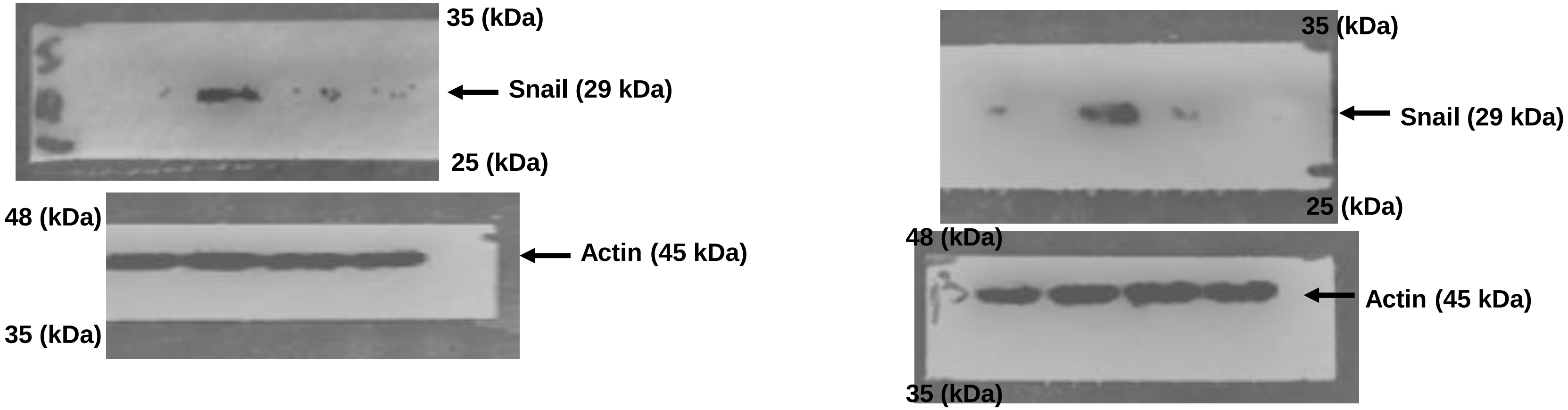

Legend
1: EGF untreated
2: EGF treated
3: EGF treated with catechol 5 μM
4: EGF treated with catechol 10 μM

Fig. S4

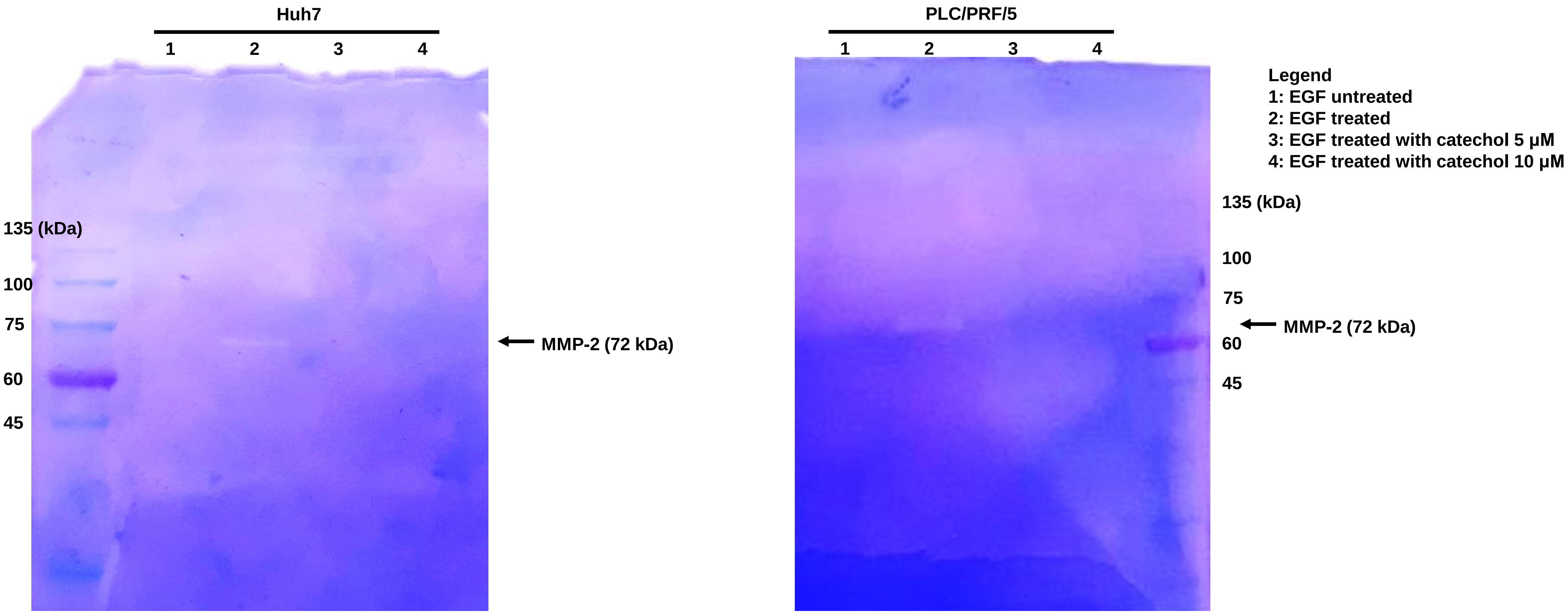
Huh7
1
2
3
4
135 (kDa)
100
75
60
45
MMP-2 (72 kDa)
PLC/PRF/5
1
2
3
4
Legend
1: EGF untreated
2: EGF treated
3: EGF treated with catechol 5 μM
4: EGF treated with catechol 10 μM
135 (kDa)
100
75
MMP-2 (72 kDa)
60
45

Fig. S5

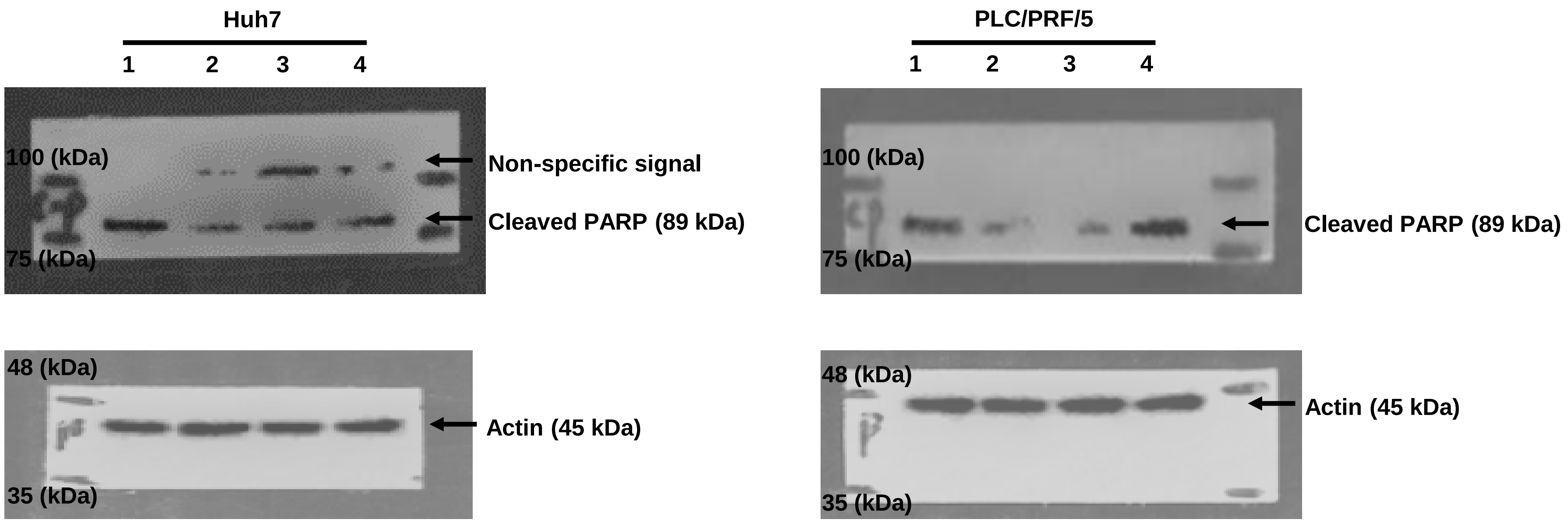

Legend
1: EGF untreated
2: EGF treated
3: EGF treated with catechol 5 μM
4: EGF treated with catechol 10 μM

Fig. S6

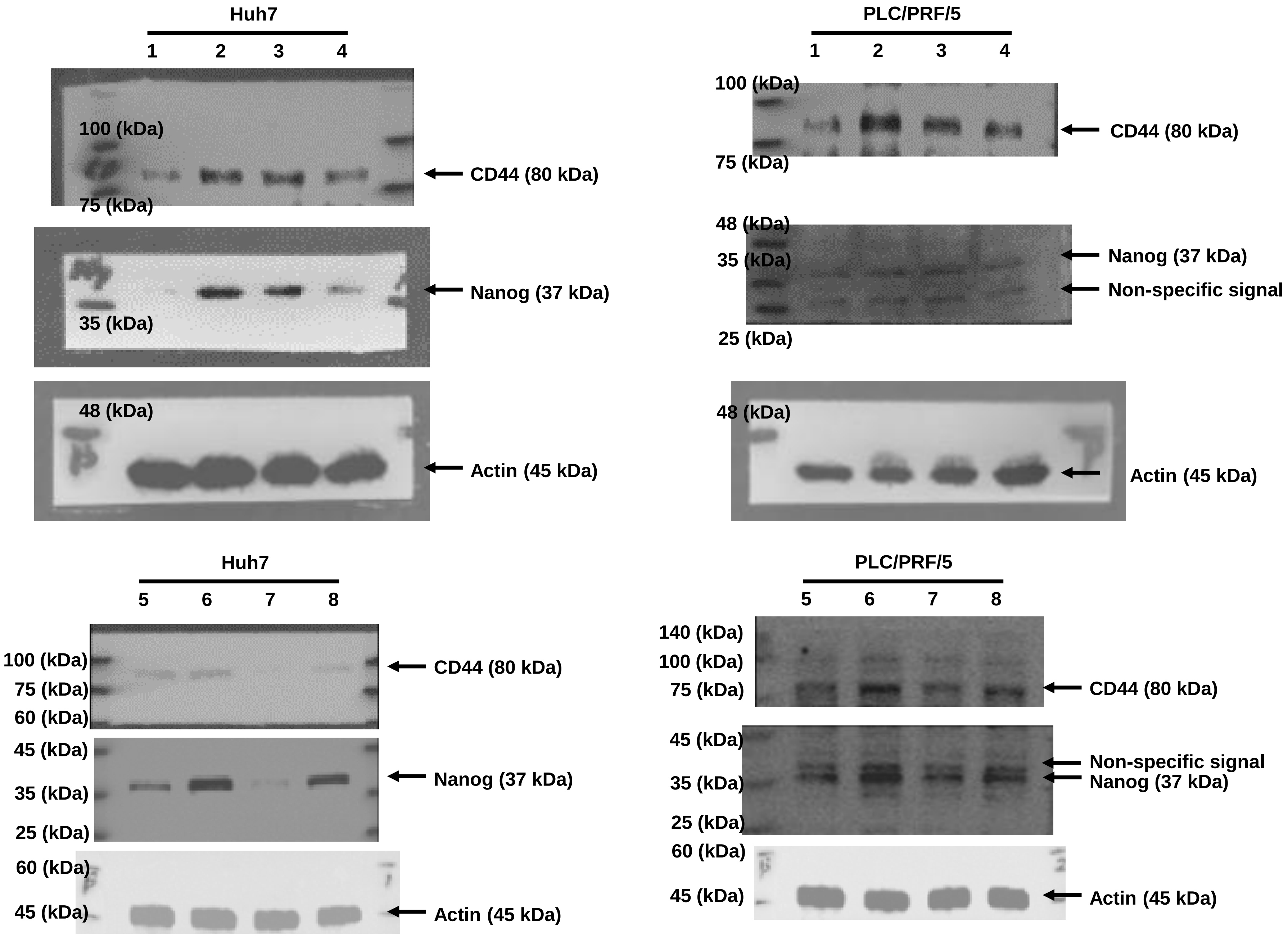

Legend
1: EGF untreated
2: EGF treated
3: EGF treated with catechol 5 µM
4: EGF treated with catechol 10 µM

5: EGF untreated
6: EGF treated
7: EGF treated with canertinib 10 µM
8: EGF treated with catechol 10 µM

Fig. S7

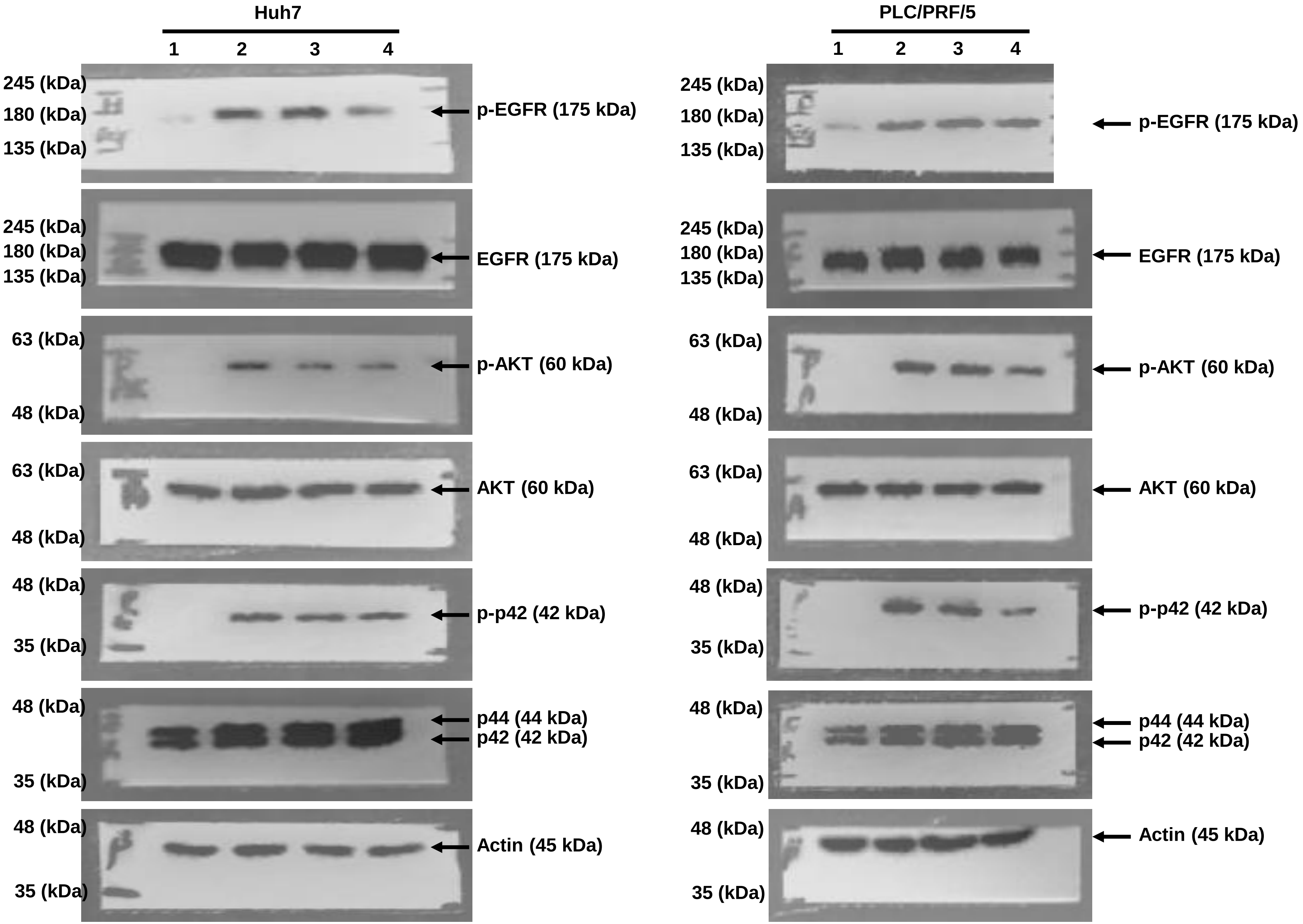

Legend
1: EGF untreated
2: EGF treated
3: EGF treated with catechol 5 μM
4: EGF treated with catechol 10 μM

Fig. S8
